# Red blood cell transfusion induces abnormal HIF-1α response to cytokine storm after adult cardiac surgery

**DOI:** 10.1038/s41598-021-01695-4

**Published:** 2021-11-15

**Authors:** Emma Viikinkoski, Juho Jalkanen, Jarmo Gunn, Tuija Vasankari, Joonas Lehto, Mika Valtonen, Fausto Biancari, Sirpa Jalkanen, K. E. Juhani Airaksinen, Maija Hollmén, Tuomas O. Kiviniemi

**Affiliations:** 1 Heart Center, Turku University Hospital and University of Turku, Hämeenkatu 11, 20521 Turku, Finland; 2 MediCity Research Laboratory, Department of Microbiology and Immunology, University of Turku, Tykistönkatu 6A, 20520 Turku, Finland; 3 Intensive Care Unit, Turku University Hospital and University of Turku, Hämeenkatu 11, 20521 Turku, Finland; 4 Brigham and Women’s Hospital, Harvard Medical School, 60 Fenwood Rd, BTM 7012, Boston, MA 02115 USA; 5 Heart and Lung Center, Helsinki University Hospital, Helsinki, Finland; 6 Research Unit of Surgery, Anesthesiology and Critical Care, Faculty of Medicine, University of Oulu, Oulu, Finland; 7 Heart Center, Turku University Hospital and University of Turku, POB 52, 20521 Turku, Finland

**Keywords:** Interleukins, Predictive markers

## Abstract

Patients undergoing cardiac surgery develop a marked postoperative systemic inflammatory response. Blood transfusion may contribute to disruption of homeostasis in these patients. We sought to evaluate the impact of blood transfusion on serum interleukin-6 (IL-6), hypoxia induced factor-1 alpha (HIF-1α) levels as well as adverse outcomes in patients undergoing adult cardiac surgery. We prospectively enrolled 282 patients undergoing adult cardiac surgery. Serum IL-6 and HIF-1α levels were measured preoperatively and on the first postoperative day. Packed red blood cells were transfused in 26.3% of patients (mean 2.93 ± 3.05 units) by the time of postoperative sampling. Postoperative IL-6 levels increased over 30-fold and were similar in both groups (p = 0.115), whilst HIF-1α levels (0.377 pg/mL vs. 0.784 pg/mL, p = 0.002) decreased significantly in patients who received red blood cell transfusion. Moreover, greater decrease in HIF-1α levels predicted worse in-hospital and 3mo adverse outcome. Red blood cell transfusion was associated with higher risk of major adverse outcomes (stroke, pneumonia, all-cause mortality) during the index hospitalization. Red blood cell transfusion induces blunting of postoperative HIF-1 α response and is associated with higher risk of adverse thrombotic and pulmonary adverse events after cardiac surgery.

**Clinical Trial Registration** ClinicalTrials.gov Identifier: NCT03444259.

## Introduction

Transfusion of red blood cells (RBC) increases oxygen delivery in patients with severe perioperative anemia. However, RBC transfusion is associated with higher risk of stroke and all-cause mortality in patients undergoing cardiac surgery^1–4^. Hypoxia induced factor-1 alpha (HIF-1α) is a key transcription factor that helps the body to adapt to inflammation caused by hypoxia^5^. In a small pilot study of six patients undergoing surgical repair of the abdominal aorta, we showed that aortic cross-clamping was associated with an increase of HIF-1α unless the patient received RBC transfusion^6^. In the present study, we sought to evaluate the systemic inflammatory response, HIF-1α levels and the related clinical outcome in patients undergoing adult cardiac surgery.

## Results

### Patient characteristics

**Figure 1:**
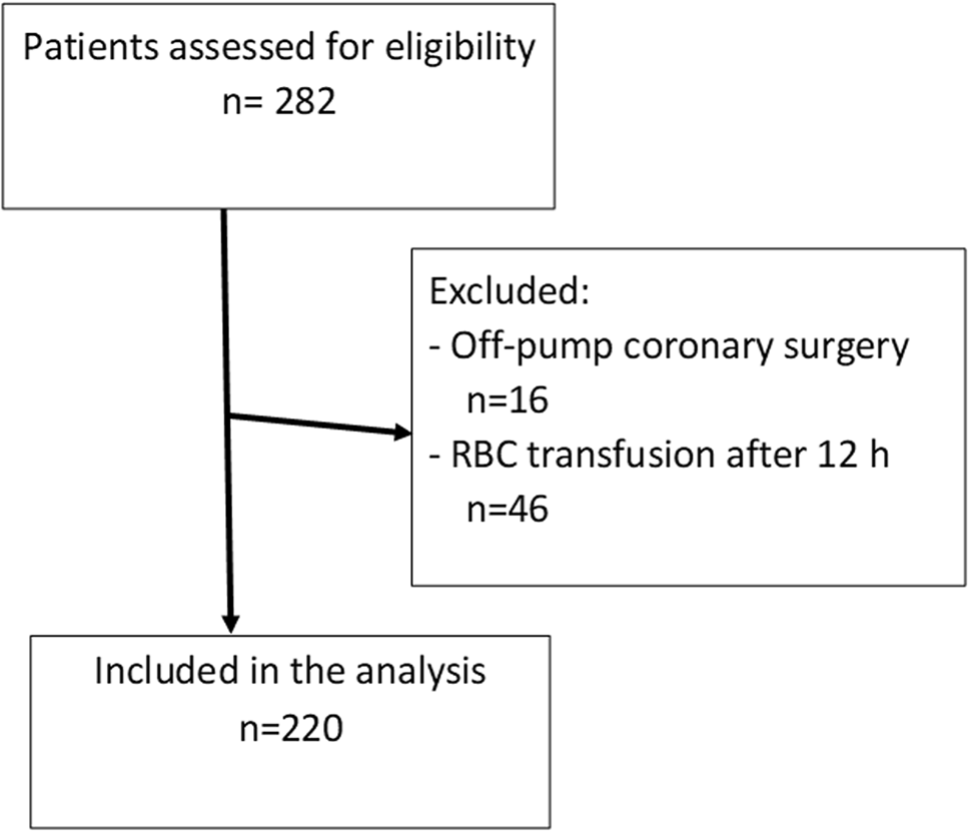
Flow chart of eligibility.

Overall, 282 patients underwent adult cardiac surgery and were included in this analysis (Fig. 1). Most operations were elective (83.0%). Four patients (1.4%) underwent emergency or salvage operation. During the index hospitalization 116/266 (43.6%) patients received RBC transfusion. Within the first 12 postoperative hours and before the second blood sample was obtained, 70/266 (26.3%) patients received RBC transfusion (45 patients received 1–2 RBC units and 25 received more than 2 RBC units). Mean transfusion amount was 2.93 ± 3.05 units among those who received RBC. Patients who received RBC transfusion after the allotted 12-h period (n = 46/266) were excluded from further analyses.

The baseline and perioperative characteristics of patients who received or not RBC transfusion within the first 12 perioperative hours are summarized in Tables 1 and 2. Patients with RBC were more often female, older, had history of congestive heart failure, and treatment for type I diabetes, hypothyroidism and hypertension. Patients who received RBC transfusion during the first 12 h had longer operation time and longer stay at the intensive care unit (ICU) after index operation. To further adjust for baseline differences, a propensity score was calculated by logistic regression. The matching caliper chosen was 0.05. One-to-one propensity score matching provided 43 pairs with similar baseline and operative characteristics except for some difference in the use of treatment for hypertension and BMI.

**Table 1:** Baseline characteristics of the study cohorts.

	RBC transfusion	No RBC transfusion	p	RBC transfusion PSM	No RBC transfusion PSM	p
	n = 70	n = 150		n = 43	n = 43	
Age	70.0 (65.0–76.0)	65.0 (56.0–71.0)	0.001	69.0 (65.0–76.0)	69.0 (62.0–76.0)	0.647
BMI	26.1 (24.0–30.0)	28.5 (± 4.5)	0.011	26.5 (± 4.6)	29.0 (± 4.7)	0.016
Preoperative eGFR	64.5 (± 22.2)	78.1 (67.1–89.4)	< 0.001	71.7 (± 21.0)	70.3 (± 14.6)	0.713
Female	28 (40.0%)	19 (12.7%)	< 0.001	12 (27.9%)	10 (23.3%)	0.621
Treatment for dyslipidemia	52 (74.3%)	97 (64.7%)	0.155	21 (72.1%)	32 (74.4%)	0.808
Treatment for diabetes II	15 (21.4%)	37 (24.7%)	0.599	8 (18.6%)	9 (20.9%)	0.787
Treatment for diabetes I	7 (10.0%)	4 (2.7%)	0.040	3 (7.0%)	1 (2.3%)	0.616
Treatment for hypertension	64 (91.3%)	109 (72.7%)	0.002	41 (95.3%)	32 (74.4%)	0.007
Heart failure	16 (22.9%)	10 (6.7%)	0.001	4 (9.3%)	3 (7.0%)	1.000
Preoperative atrial fibrillation	16 (22.9%)	30 (20.0%)	0.627	8 (18.6%)	6 (14.0%)	0.559
Coronary artery disease	49 (70.0%)	90 (60.0%)	0.152	27 (62.8%)	29 (67.4%)	0.651
Previous myocardial infarction	18 (25.7%)	33 (22.0%)	0.543	8 (18.6%)	13 (30.2%)	0.209
Recent myocardial infarction	14 (20.0%)	16 (10.7%)	0.060	5 (11.6%)	9 (20.9%)	0.243
Prior PCI	11 (15.7%)	19 (12.7%)	0.540	8 (18.6%)	8 (18.6%)	1.000
Prior stroke	8 (11.4%)	14 (9.3%)	0.629	3 (7.0%)	1 (2.3%)	0.616
Carotid artery disease	3 (4.3%)	1 (0.7%)	0.096	2 (4.7%)	1 (2.3%)	1.000
Peripheral artery disease	4 (5.7%)	3 (2.0%)	0.213	1 (2.3%)	1 (2.3%)	1.000
Pulmonary artery hypertension	26 (37.1%)	41 (27.3%)	0.141	14 (32.6%)	13 (30.2%)	0.816
Chronic lung disease	9 (12.9%)	12 (8.0%)	0.253	5 (11.6%)	3 (7.0%)	0.713
Active smoking	13 (18.6%)	25 (16.9%)	0.760	6 (14.0%)	3 (7.3%)	0.484
Ex-smoker	30 (42.9%)	64 (43.2%)	0.957	22 (51.2%)	17 (41.5%)	0.373
Obstructive sleep apnea	6 (8.6%)	13 (8.7%)	0.981	3 (7.05)	5 (11.6%)	0.713
Any malignancy	14 (20.0%)	17 (11.3%)	0.085	8 (18.6%)	6 (14.0%)	0.559
Chronic dialysis	3 (4.3%)	0 (0.0%)	0.030	1 (2.4%)	0(0.0%)	0.494
Hypothyroidism	14 (20.0%)	6 (4.0%)	< 0.001	6 (14.0%)	4 (9.3%)	0.501

Continuous variables are reported as median and interquartile range or mean and standard deviation. Categorical variables are reported as counts and percentages.

*BMI* body mass index, *CABG* coronary artery bypass grafting, *CPAP* continuous positive airway pressure, *eGFR* estimated glomerular filtration rate, *LED* discoid lupus erythematosus, *PCI* percutaneous coronary intervention; *RBC* red blood cell, *SLE* systemic lupus erythematosus, *TAVR* transcatheter aortic valve replacement.

**Table 2:** Operative characteristics of the study cohorts.

	RBC transfusion	No RBC transfusion	p	RBC transfusion PSM	No RBC transfusion PSM	p
	n = 70	n = 150		n = 43	n = 43	
Elective operation	55 (78.6%)	133 (88.7%)	0.048	37 (86.0%)	39 (90.7%)	0.501
Urgent operation	13 (18.6%)	16 (10.7%)	0.106	4 (9.3%)	4 (9.3%)	1.000
Emergency/salvage operation	2 (2.9%)	1 (0.7%)	0.238	2 (4.7%)	0 (0.0%)	0.494
**Type of procedure**						
AVR biological prosthesis/mechanical prosthesis	21 (30.0%)	48 (32.0%)	0.766	12 (27.9%)	16 (37.2%)	0.357
CABG	49 (70.0%)	87 (58.0%)	0.088	27 (62.8%)	29 (67.4%)	0.651
Isolated CABG	33 (47.1%)	69 (46.0%)	0.874	19 (44.2%)	23 (53.5%)	0.388
MVP	3 (4.3%)	15 (10.0%)	0.150	2 (4.7%)	3 (7.0%)	1.000
MVR	7 (10.0%)	2 (1.3%)	0.005	5 (11.6%)	0 (0.0%)	0.055
Surgery on the ascending aorta	6 (8.6%)	17 (11.3%)	0.533	4 (9.3%)	1 (2.3%)	0.360
David procedure	0 (0.0%)	4 (2.7%)	0.309	0 (0.0%)	0 (0.0%)	–
Bentall-DeBono procedure	1 (1.4%)	10 (6.7%)	0.180	1 (2.3%)	1 (2.3%)	1.000
Maze procedure	2 (2.9%)	4 (2.7%)	1.000	1 (2.3%)	0 (0.0%)	1.000
Pericardiectomy	2 (2.9%)	1 (0.7%)	0.238	1 (2.3%)	1 (2.3%)	1.000
LAA closure	11 (15.7%)	17 (11.3%)	0.346	6 (14.0%)	3 (7.0%)	0.483
Other procedures	1 (1.4%)	3 (2.0%)	1.000	1 (2.3%)	1 (2.3%)	1.000
**Indications for CABG**						
Stable angina	22 (31.4%)	55 (36.7%)	0.448	15 (34.9%)	18 (41.9%)	0.506
Unstable angina	10 (14.3%)	14 (9.3%)	0.272	5 (11.6%)	2 (4.7%)	0.433
NSTEMI	17 (24.3%)	16 (10.7%)	0.008	7 (16.3%)	9 (20.9%)	0.579
STEMI	0 (0.0%)	2 (1.3%)	1.000	0 (0.0%)	0 (0.0%)	–
Operation length (min)	246 (209–285)	225 (197–250)	0.006	238 (210–292)	233 (± 39)	0.346
Aortic cross-clamping time (min)	89 (74–112)	89 (72–106)	0.363	89 (71–102)	91 (75–111)	0.678
Cardiopulmonary bypass time (min)	115 (102–145)	114 (94–138)	0.136	114 (93–138)	124 (± 32)	0.445
Delayed ventilation*	10 (14.3%)	0 (0.0%)	< 0.001	7 (16.3%)	0 (0.0%)	0.012
Duration of mechanical ventilation (hours)	11.5 (6.4–20.6)	5.0 (4.0–7.0)	< 0.001	15.0 (8.5–21.5)	5.5 (4.5–8.0)	< 0.001
**Blood products administered within 12 h**						
Packed red blood cells	70 (100.0%)	0 (0.0%)	< 0.001	43 (100.0%)	0 (0.0%)	< 0.001
Fresh frozen plasma	27 (38.6%)	21 (14.0%)	< 0.001	18 (41.9%)	8 (18.6%)	0.019
Platelets	31 (44.3%)	14 (9.3%)	< 0.001	21 (48.8%)	1 (2.3%)	< 0.001
**Intensive care unit variables**						
Length of ICU stay (hours)	25 (22–47)	23 (22–24)	< 0.001	24 (22–47)	23 (22–24)	0.006
Intravenous fluids (mL, 12 h)	3878 (2543–5849)	2752 (2419–3290)	< 0.001	4249 (2381–6081)	3060 (± 1017)	0.005
Chest drain output (mL, 12 h)	565 (260–1139)	368 (290–480)	0.001	600 (320–1195)	410 (295–530)	0.009
Diuresis (mL, 12 h)	1860 (1390–2520)	1960 (1655–2348)	0.605	1820 (1360–2470)	2055 (± 749)	0.678

Continuous variables are reported as median and interquartile range or mean and standard deviation. Categorical variables are reported as counts and percentages.

*AVR* aortic valve replacement, *CABG* coronary artery bypass grafting, *ECMO* extracorporeal membrane oxygenation, *IABP* intra-aortic balloon pump, *ICU* intensive care unit, *LAA* left atrial appendix, *MVP* mitral valve repair, *MVR* mitral valve replacement; *NSTEMI* non-ST-elevation myocardial infarction, *RBC* red blood cell, *STEMI* ST-elevation myocardial infarction.

*Delayed ventilation > 24 h.

### IL-6 and HIF-1α response

**Figure 2:**
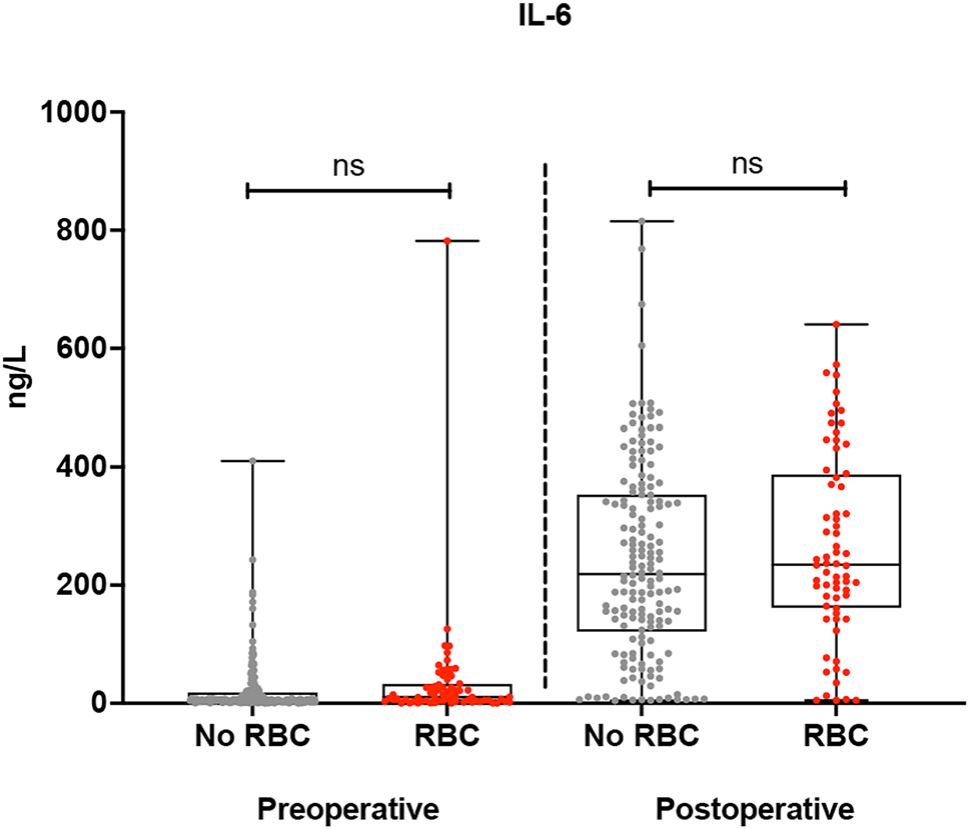
Pre- and postoperative interleukine-6 (IL-6) cytokine levels (ng/L) in with and without red blood cell transfusion (RBC) after adult cardiac surgery. The vertical axis represents IL-6 levels (ng/L). The horizontal axis represents patients who received or not RBC transfusion preoperatively and postoperatively.

**Figure 3:**
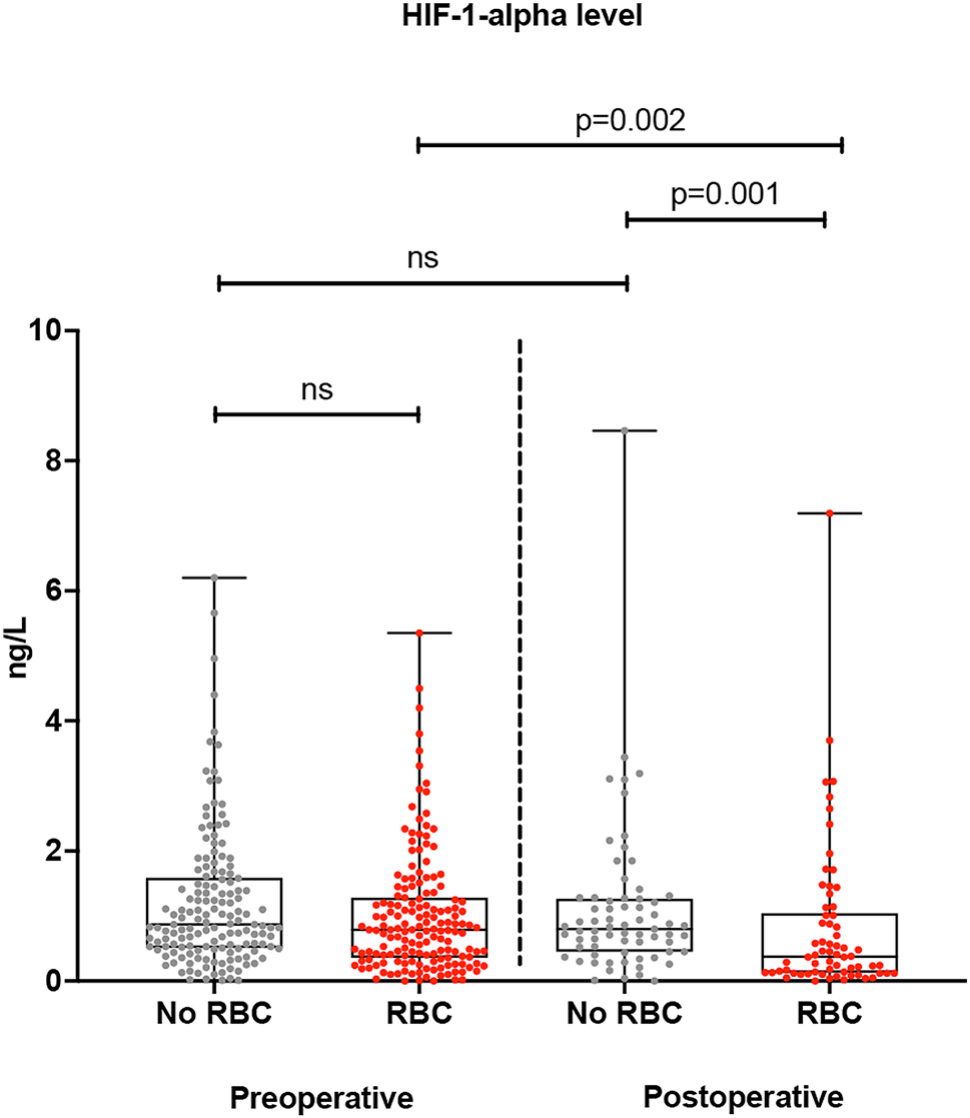
Pre- and postoperative hypoxia-inducible factor 1 alpha (HIF-1-α) levels (ng/L) in patients with or without red blood cell transfusion (RBC) after adult cardiac surgery. The vertical axis represents HIF-1α cytokine levels (ng/L). The horizontal axis represents patients who received or not RBC transfusion preoperatively and postoperatively.

**Figure 4:**
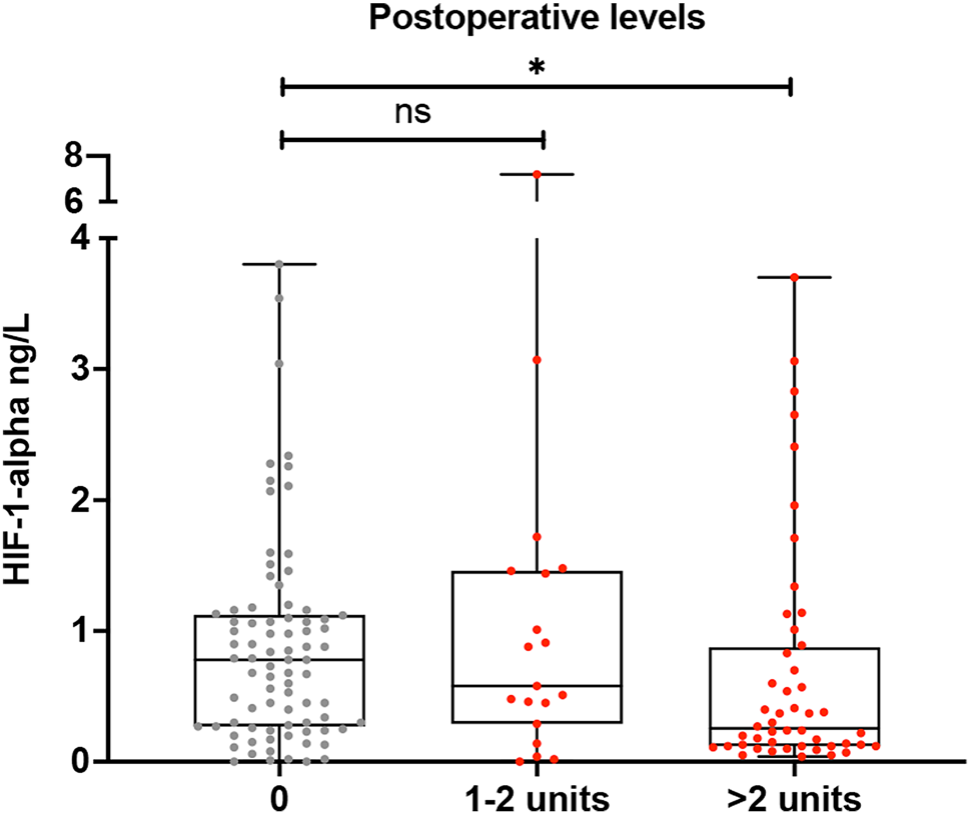
Postoperative hypoxia-inducible factor 1 alpha (HIF-1-α) levels according to the number of transfused red blood cell units. The vertical axis represents HIF-1α cytokine levels (ng/L). The horizontal axis represents red blood cell units in three groups.

As a marker of cytokine derangement, IL-6 levels were increased postoperatively over 30-fold without any difference between the study groups (236.1 pg/mL vs. 211.5 pg/mL, p = 0.130) (Fig. 2). Interestingly, patients who received RBC transfusion had an insufficient adaptive response to hypoxia—measured as significantly decreased HIF-1α levels during the first 12 h postoperatively—compared to those who did not receive any blood (0.377 pg/mL vs. 0.786 pg/mL, p = 0.002) (Fig. 3). Giving more than two units of packed red blood cells dumped HIF-1α response even further (Fig. 4). When adjusted for transfusions over 2 units of packed red blood cells, there was a significant difference in postoperative and delta-HIF-1α levels between the study groups (p < 0.001 and p = 0.047, respectively). In multivariate regression analysis RBC transfusion considerably blunted the HIF-1α response when significant baseline characteristics were included in the model (BMI, preoperative paroxysmal atrial fibrillation, previous myocardial infarction, treatment for dyslipidemia, carotid artery disease, hypothyroidism, operation type coronary artery bypass (CABG), operation indication non-ST-elevation myocardial infarction (NSTEMI) for CABG, RBC transfusion). Results show a significant effect on HIF-1α response (F(9.205) = 3.039), p = 0.002, with R^2^ = 0.118, suggesting prediction value of 11.8% by the listed factors. Blood transfusion was found to be the highest predictor for HIF-1α response (p = 0.037) (see Supplemental Table S1). Platelet and fresh frozen plasma administration were not included in the model since they are administrated with red blood cells.

### Clinical outcomes

Clinical outcome events during hospitalization and at 90 days are summarized in Table 3. Patients who received RBC transfusion during the first 12 h perioperatively had substantially higher rates of strokes, postoperative pneumonia, de novo dialysis, and mortality during the index hospitalization as well as longer intensive care unit and hospital stay. In the PSM cohort, there were more adverse events in the RBC transfusion group and this was mainly driven by higher postoperative pneumonia and stroke rates.

**Table 3:** Clinical outcomes.

	RBC transfusion	No RBC transfusion	p	RBC transfusion PSM	No RBC transfusion PSM	p
	n = 70	n = 150		n = 43	n = 43	
**Index hospitalization**						
Death	6 (8.6%)	0 (0.0%)	0.001	4 (9.3%)	0 (0.0%)	0.116
New-onset atrial fibrillation*	25 (46.3%)	43 (35.8%)	0.191	14 (40.0%)	20 (54.1%)	0.233
Stroke	7 (10.0%)	1 (0.7%)	0.002	5 (11.6%)	0 (0.0%)	0.055
TIA	0 (0.0%)	0 (0.0%)	–	0 (0.0%)	0 (0.0%)	–
Postoperative pneumonia	8 (11.4%)	2 (1.3%)	0.002	6 (14.0%)	0 (0.0%)	0.026
Deep sternal wound infection/mediastinitis	2 (2.9%)	2 (1.3%)	0.594	1 (2.3%)	1 (2.3%)	1.000
Reoperation for bleeding	16 (22.9%)	1 (0.7%)	< 0.001	13 (30.2%)	1 (2.3%)	< 0.001
De novo dialysis	3 (4.3%)	0 (0.0%)	0.031	2 (4.7%)	0 (0.0%)	0.494
Major adverse events†	16 (22.9%)	3 (2.0%)	< 0.001	11 (25.6%)	0 (0.0%)	< 0.001
Length of hospital stay (days)	10 (7–16)	7 (7–9)	< 0.001	9 (7–15)	7 (7–9)	0.012
**Heart rhythm at the time of discharge**^**‡**^						
Sinus rhythm	52 (80.0%)	114 (76.0%)	0.521	34 (85.0%)	29 (67.4%)	0.062
Atrial fibrillation	12 (18.5%)	32 (21.3%)	0.632	6 (15.0%)	13 (30.2%)	0.099
Other	1 (1.5%)	4 (2.7%)	1.000	0 (0.0%)	1 (2.3%)	1.000
**Events within 90 days**						
NOAF	26 (49.1%)	48 (40.3%)	0.286	15 (44.1%)	20 (54.1%)	0.403
NOAF after index hospitalization	1 (3.6%)	5 (6.6%)	1.000	7 (18.4%)	10 (23.3%)	0.594
Permanent anticoagulation	16 (25.4%)	32 (21.5%)	0.533	8 (21.1%)	12 (27.9%)	0.475
Myocardial infarction	0 (0.0%)	1 (0.7%)	1.000	0 (0.0%)	0 (0.0%)	–
Stroke and TIA	9 (13.8%)	3 (2.0%)	0.001	6 (15.0%)	1 (2.3%)	0.052
Death	9 (12.9%)	0 (0.0%)	< 0.001	5 (11.6%)	0 (0.0%)	0.055

Continuous variables are reported as median and interquartile range or mean and standard deviation. Categorical variables are reported as counts and percentages.

*NOAF* New-onset atrial fibrillation, *TIA* transient ischemic attack.

*Patients with preoperative atrial fibrillation or atrial flutter (n = 60) were excluded from the variable.

^†^Major adverse event includes stroke, TIA, postoperative pneumonia and death during index hospitalization.

^‡^Other: pacemaker or junctional rhythm.

Lower decrease in HIF-1α levels—as an indication for minor blunting—predicted better composite outcome of all-cause mortality, stroke, transient ischemic attack (TIA), pneumonia, mediastinitis, acute dialysis or reoperation for bleeding during index hospitalizaton (OR 0.42, 95% CI 0.21–0.84) and at 3mo follow-up (OR 0.50, 95% CI 0.26–0.98) in a multivariable logistic regression model including age and gender as covariates in the overall series and in the PSM cohort.

## Discussion

The main finding of this study was that RBC transfusion may significantly reduce the response to hypoxia by inhibiting HIF-1α elevation in the cytokine storm secondary to surgical stress and the use of cardiopulmonary bypass. RBC transfusion significantly increases the risk of major adverse events after cardiac surgery^7–9^.

RBC use was associated with high rates of in-hospital adverse events. Risk for stroke/TIA was over 16-fold and every tenth patient with RBC use had a stroke/TIA. The clinical setting and baseline differences in groups need to be taken into account when interpreting the results. Patients who received RBC transfusion were frailer at baseline. In PSM analysis almost all transfusion patients were on medication for hypertension, and we observed no difference in the usage of betablockers, ACE-inhibitors/angiotensin receptor blockers or calcium-channel blockers between transfusion groups. Differences in clinical findings during hospitalization indicate more straining surgeries for RBC transfusion patients. In multivariate logistic regression and propensity score analysis we showed that RBC was substantial predictor for these thrombotic events unlike other baseline and perioperative variables. Larger scale studies with similar baseline and clinical presentation have previously reported even more pronounced role for RBC transfusion with worse outcome and mortality^10,11^.

Our findings are in line with previous studies on transfusions and postoperative stroke occurrence^1,2,4^. We also found stroke risk to be higher if patient was transfused more than two units^12^. Etiology of post-operative strokes is often cardioembolic after open-heart surgery^13^ and a great share is atrial fibrillation-related and derived from the left atrial appendage. Risk for pneumonia was almost tenfold and only renal impairment in addition to RBC was its predictor. These results highlight the findings of abnormal thrombotic and inflammatory milieu after RBC in postoperative setting.

RBC transfusion effect on worsen outcome has been suggested to be related to RBC quality after longer storage time^14,15^. We provide observational mechanistic hypothesis for RBC-related increase in adverse outcomes. Cardiopulmonary bypass and open-heart surgery induce hypoxia as well as a drastic cytokine storm^16^. As a sign of this storm, IL-6 levels rose 30-fold postoperatively in both groups. HIF-1α—found ubiquitously—helps the body to adapt to inflammation caused by hypoxia^5^. We show that blunted HIF-1α was most common in patients who received RBC. To assess whether this finding was independent of other baseline and perioperative factor, we performed multiple sensitivity analyses where RBC remained as the most influential predictor for the abnormal response. With transfusion of more than two units of RBC, post-operative HIF-1α was more pronounced. Greater decrease of HIF-1α was associated with adverse in-hospital and 3mo follow-up outcomes.

Blunted HIF-1α response likely disrupts body’s adaptation to hypoxic stimuli. HIF-1α is a transcription factor which induces transcription of more than 60 genes in hypoxia^17^ including the de novo synthesis of CD73^18^. Extra-cellular CD73 (ecto-5'-nucleotidase/NT5E)-derived adenosine production is one of the key pathways in attenuating hypoxia-induced inflammation^19,20^ and protecting several central organs in the acute hypoxia^21^. CD73 is a glycosyl-phosphatidylinositol expressing cell surface enzyme found on most human tissues, such as endothelial cells and immune cells. Recent studies have suggested CD73 has a role in myocardial infarction recovery^22^, central nervous system protection^23^, and control of alveolar permeability in hypoxia and vascular leak^24^. To our knowledge, we provide association of RBC with blunted HIF-1α response as well as increased rates of thrombotic and lung injury endpoints for the first time.

The strength of our study was that it was a prospective cohort study with a consecutive patient enrollment. We included in our final analysis patients who had open-heart surgery under CPB and who received packed red blood perioperatively excluding patients who needed red blood cells later the appointed 12 h during index hospitalization. Patients’ operation types did not differ between groups. There were a few limitations in our study. Baseline characteristics differed between groups since patients who needed more often packed red blood were older, female and had more comorbidities. Female sex has been shown to act as an independent risk factor for red blood cell transfusion^25^. Obviously, patients received RBC for clinical indications such as anemia and perioperative bleeding and the decision on RBC use was always at the treating anesthesiologist’s discretion. We need to consider that RBC transfusion may be a result not a cause for complications. We tackled this challenge using propensity score matching. We registered 33 postoperative outcome events in PSM group and 48 outcome events in total. Since new-onset atrial fibrillation is very common after cardiac surgery, we did not add this covariate in our regression model. In multivariate logistic regression we could only use few explanatory variables due to statistical constraints. We chose age and gender as the other covariates as they are known predictors of worse postoperative outcome. RBC transfusion was nominated an explanatory covariate for postoperative outcome mirroring previous studies. There are multiple studies on red blood cell transfusion related worse postoperative outcome and transfusion has been shown to act an independent predictor^10,11^. Relatively small sample size as well as observational (yet, prospective) setting are other limitations of this study, and therefore, these findings should be viewed as hypothesis generating. One limitation was that pre- and postoperative blood samples were taken not more than 24 h apart and packed red blood cells were given 12 h perioperatively which meant measured cytokine response happened in a short window.

This study has clinical implications. Decision on RBC use needs to be balanced between its anticipated benefits and harms. This study supports the view that we may need to consider stricter threshold limit for transfusions^26,27^. Intraoperative autologous blood donation (IAD) has been recently shown to reduce transfusion need and reduction in postoperative complications^28^*.* Moreover, hypoxia-inducible inflammation and impaired hypoxia related repair mechanisms require further research and could serve as a target for operative preconditioning and better patient outcome.

In conclusion, the present findings provide evidence that the use of RBC transfusion disrupts the adaptation to cytokine storm and hypoxia and is associated with adverse thrombotic and inflammatory events after adult cardiac surgery.

## Methods

### Patient selection

The present analysis includes a prospective cohort of 282 consecutive patients who underwent adult cardiac surgery from February 2016 to September 2017 at the Heart Center of the Turku University Hospital, Turku, Finland. Our Institution is a University and tertiary referral hospital for a population of 876,000 inhabitants of Southwest Finland. These patients are participants to the CAREBANK study (Cardiovascular Research Consortium—a Prospective Project to Identify Biomarkers of Morbidity and Mortality in Cardiovascular Interventional Patients), an on-going Finnish prospective cohort study of patients undergoing adult cardiac surgery at the Turku University Hospital, Turku, Finland (ClinicalTrials.gov Identifier: NCT03444259), first posted on 23/02/2018. The cohort consists of all the recruited patients who have undergone open-heart surgery and have 90-day follow-up data by the time of immunological analysis by December 2017. Blood samples from these patients were prospectively obtained pre- and postoperatively. Follow-up data was collected prospectively both by individual contacts by phone calls using a structured questionnaire, and from hospital records at prespecified time points (3, 12 and 24 months). This study focused on in-hospital outcomes and 90-day outcomes on the assumption perioperative blood transfusion related outcomes were most likely seen at these time periods. Mortality data was obtained from the nationwide registry Statistics Finland. The CAREBANK has received approval from the Ethical Committee of the Hospital District of Southwestern Finland and adheres to the Declaration of Helsinki as revised 2002. CAREBANK data was monitored by an independent third party. Written informed consent was obtained from the study subjects. The data that support the findings of this study are available from the corresponding author upon reasonable request.

### Patient serum samples

Consecutive blood samples were collected preoperatively and on the morning of the first postoperative day. Pre- and postoperative serum was centrifuged from the whole blood and ethylenediaminetetraacetic acid plasma. The first samples were obtained on the morning time after fasting or at the emergency setting. The second blood samples were obtained on the first postoperative morning with patients still fasting. The serum samples were labeled and stored at – 70 °C until analyses. The analyses for all 282 samples were done in three separate days and the three persons assigned to do the cytokine analyses were unaware of patients’ procedures and outcomes. IL-6 and HIF-1α analyses were done with the enzyme-linked immunosorbent assay according to manufacturer’s instructions (Elabscience, Houston, Texas, USA). The optical density values were analyzed using Tecan Infinite M200 and Magellan 7.2. software for Microsoft Windows (Tecan Group, Männedorf, Switzerland).

### Outcomes

The primary outcome of this study was the evaluation of cytokine IL-6 and HIF-1α levels in respect with RBC transfusion during surgery and/or within 12 h from surgery. The secondary outcomes were postoperative complications during the index hospitalization after open-heart surgery, new onset atrial fibrillation at 90 days, three-month stroke, and morbidity.

The diagnosis of in-hospital new onset atrial fibrillation (NOAF) was confirmed by a 12-lead ECG recording or telemonitoring indicating an atrial fibrillation episode of 10 min or longer. Ischemic stroke was defined as a permanent focal neurological deficit adjudicated by a neurologist and confirmed via computed tomography (CT) or magnetic resonance imaging (MRI). Only ischemic strokes considered definite by the treating neurologist or physician were included in the present study. Pneumonia was defined as symptoms of infection (fever, malaise), elevation of C-reactive protein (CRP) or leucocyte count (excluding leg wound infection), and/or imaging finding consistent with infection (X-ray, CT, magnetic resonance imaging MRI or ultrasound) and/or evident purulent wound secretion or abscess. Mediastinitis was defined as symptoms of infection (fever, malaise), elevation of CRP or leucocyte count (excluding leg wound infection), and/or imaging finding consistent with infection at the mediastinal area and without signs of alternative diagnoses with similar presentation. Re-operation for bleeding was defined as re-sternotomy because of bleeding.

### Statistical analysis

Statistical analyses were performed using IBM SPSS Statistics version 25 (SPSS Inc., Chicago, IL, USA). Continuous variables are reported as median and inter-quartile range (IQR) or mean and standard deviation (SD). Categorical variables are reported as counts and percentages. The Shapiro–Wilk test of normality was performed to all baseline characteristics. The Chi-square test and Fischer’s exact test were used to evaluate the difference in categorical variables and outcomes whilst continuous variables were evaluated using independent sample T-test or Mann–Whitney-U test. Logistic regression was performed to identify risk factors associated with decreased postoperative HIF-1α levels. Multivariate logistic regression models were performed by including variables of relevance with p-value < 0.05 in the univariable analyses. HIF-1α levels were log-transformed for logistic regression analysis. Statistical significance was set at p < 0.05 in multivariate analysis. Propensity score matching (PSM) was conducted to better evaluate endpoint events and reduce bias. This study was exploratory in nature and a formal sample size calculation was not carried out. We anticipated that a sample of 300 patients would be reasonable to show consistent change in IL-6 and HIF-1α levels pre and post operation.

## Supplementary Information

Supplementary Tables.
