# Supplementary material for: Red blood cell transfusion induces abnormal HIF-1α response to cytokine storm after adult cardiac surgery
Source: Sci Rep. 2021 Nov 15;11:22230. doi: 10.1038/s41598-021-01695-4 (PMC8592994; doi:10.1038/s41598-021-01695-4)
Supplement: Supplementary file 1 — Supplementary Tables. [file 41598_2021_1695_MOESM1_ESM.pdf]

SUPPLEMENTAL MATERIAL

Red blood cell transfusion induces abnormal HIF-1α response to cytokine storm after adult cardiac surgery

Emma Viikinkoski, MD[1], Juho Jalkanen, MD, PhD[2], Jarmo Gunn, MD, PhD, Prof[1], Tuija Vasankari, RA[1], Joonas Lehto, MD, PhD[1], Mika Valtonen, MD, PhD,[3] Fausto Biancari, MD, PhD, Prof,[1,5,6] Sirpa Jalkanen, MD, PhD, Prof[2], K E Juhani Airaksinen, MD, PhD, Prof[1], Maija Hollmén, PhD, Doc[2], and Tuomas O Kiviniemi, MD, PhD, FESC[1,4] *

Supplemental Table I. Association between clinical and operative variables and postoperative logHIF-1α (N=262). Linear regression.

	Univariate analysis					Multivariate analysis	
	**B**	**SE**	**β**	**t**	**P**	**β**	**P**
Age	-.004	.003	-.073	-1.173	.242	NP	
Preoperative eGFR	.002	.002	.079	1.265	.207	NP	
BMI	.017	.008	.139	2.248	.025	.062	.363
Female	-.062	.088	-.044	-.708	.480	NP	
Preoperative atrial fibrillation	-.001	.085	.000	-.008	.994	NP	
Permanent	-.118	.123	-.059	-.958	.339	NP	
Paroxysmal	.345	.171	.125	2.024	.044	.038	.571
Coronary artery disease	.080	.073	.068	1.103	.271	NP	
Previous myocardial infarction	.207	.082	.155	2.538	.012	.128	.190
Recent myocardial infarction	.196	.101	.120	1.944	.053	NP	
Treatment for dyslipidemia	.173	.075	.142	2.310	.022	.132	.112
Prior PCI	.115	.170	.042	.676	.500	NP	
Prior CABG	-	-	-	-	-	NP	
Prior aortic valve surgery	-.244	.219	-.069	-1.111	.268	NP	
Prior mitral valve surgery	.113	.407	.017	.278	.781	NP	
Previous TAVR	.224	.575	.024	.390	.697	NP	
Previous percutaneous mitral valve procedure	-	-	-	-	-	NP	
Treatment for diabetes II	.058	.082	.044	.710	.478	NP	
Treatment for diabetes I	.196	.169	.072	1.158	.248	NP	
Treatment for hypertension	-.011	.089	-.008	-.127	.899	NP	
Heart failure	.072	.102	.044	.703	.482	NP	
Previous stroke	.143	.114	.077	1.251	.212	NP	
Carotid artery disease	-.565	.217	-.159	-2.604	.010	-.112	.099
Peripheral artery disease	-.066	.177	-.023	-.374	.708	NP	
Active endocarditis	.544	.406	.083	1.340	.182	NP	
Previous endocarditis	-.105	.333	-.020	-.315	.753	NP	
Hepatic cirrhosis	.056	.575	.006	.097	.923	NP	

Pulmonary artery hypertension	.056	.076	.046	.742	.459	NP	
Chronic lung disease	-.041	.113	-.023	-.367	.714	NP	
Active smoking	.170	.092	.114	1.840	.067	NP	
Ex-smoker	-.037	.070	-.033	-.534	.594	NP	
Obstructive sleep apnea	.004	.123	.002	.035	.972	NP	
CPAP mask	-.083	.163	-.031	-.507	.613	NP	
Inflammatory bowel disease	.129	.206	.039	.626	.532	NP	
Psoriatic arthropathy	.104	.237	.027	.438	.662	NP	
Skin disease	-.009	.116	-.005	-.081	.936	NP	
Seropositive rheumatoid arthritis	.183	.289	.039	.633	.527	NP	
Seronegative rheumatoid arthritis	.015	.259	.004	.057	.955	NP	
SLE/ LED	.101	.575	.011	.176	.860	NP	
Hypothyroidism	-.313	.124	-.155	-2.531	.012	-.137	.048
Hyperthyroidism	-	-	-	-	-	NP	
Malignancy	-.087	.101	-.054	-.868	.386	NP	
Active	-.344	.288	-.074	-1.192	.235	NP	
Remission	-.077	.104	-.046	-.737	.462	NP	
Poor mobility	-	-	-	-	-	NP	
Connective tissue disease	-.250	.256	-.061	-.974	.331	NP	
Chronic dialysis	.120	.286	.026	.420	.675	NP	
**Operation status:**							
Elective	-.126	.097	-.080	-1.292	.197	NP	
Urgent	.148	.101	.090	1.462	.145	NP	
Emergency and salvage	-.092	.289	-.020	-.318	.751	NP	
**Operation type:**							
AVR (bioprosthesis/ mechanical)	-.069	.075	-.057	-.929	.354	NP	
MPL	-.114	.130	-.054	-.876	.382	NP	
MVR	-.102	.185	-.034	-.550	.583	NP	
CABG	.130	.072	.112	1.809	.072	NP	
Only CABG	.166	.071	.145	2.355	.019	-.015	.865
Surgery on ascending aorta	-.067	.117	-.036	-.578	.564	NP	
DAVID procedure	-	-	-	-	-	NP	
Bentall procedure	-.074	.148	-.031	-.502	.616	NP	
MAZE procedure	.106	.220	.030	.485	.628	NP	
Pericardiectomy	-.474	.332	-.088	-1.428	.154	NP	
LAA closure	.108	.105	.064	1.030	.304	NP	

Other procedure	-	-	-	-	-	NP	
**Indication for CABG:**							
Stable	.009	.075	.007	.116	.908	NP	
UAP	-.011	.117	-.006	-.096	.924	NP	
NSTEMI	.205	.097	.130	2.119	.035	.018	.852
STEMI	.431	.406	.066	1.061	.289	NP	
**Operation specifics:**							
Operation length (min)	3.163E-5	.000	.010	.149	.882	NP	
Aortic cross-clamping time (min)	-.002	.001	-.088	-1.412	.159	NP	
Cardiopulmonary bypass time (min)	.000	.000	-.042	-.677	.499	NP	
Cardioplegia Antegrade	-.105	.289	-.023	-.362	.717	NP	
Cardioplegia Retrograde	-.116	.076	-.095	-1.528	.128	NP	
Endoscopic vein harvest	.297	.257	.072	1.154	.250	NP	
DHCA	-.304	.287	-.066	-1.058	.291	NP	
Delayed ventilation	-.034	.122	-.017	-.275	.783	NP	
Time in ventilator (hours)	-.001	.000	-.119	-1.924	.055	NP	
ECMO	.000	.575	.000	-.001	.999	NP	
IABP treatment	.187	.258	.045	.724	.469	NP	
Noradrenalin infusion	-.144	.332	-.027	-.433	.665	NP	
Adrenalin infusion	-.150	.136	-.069	-1.107	.269	NP	
Milrinone	-.006	.087	-.004	-.064	.949	NP	
Levosimendan	.027	.087	.019	.307	.759	NP	
Caprilon	-	-	-	-	-	NP	
Coagulation factor VIIa	-.107	.407	-.016	-.262	.794	NP	
Coagulation factor VIII	-.288	.575	-.031	-.501	.617	NP	
Coagulation factor XIII	-.163	.085	-.118	-1.917	.056	NP	
Human fibrinogen	-.481	.235	-.126	-2.047	.042	NP	
**Blood products administered 12 hours perioperatively:**							
Packed red blood cells	-.241	.085	-.190	-2.830	.005	-.151	.037
Fresh frozen plasma	-.568	.080	-.405	-7.138	.000	NP	
Platelets	-.184	.082	-.139	-2.255	.025	NP	
**Intensive care unit:**							
Length of stay (hours)	.003	.005	.034	.541	.589	NP	
Total fluids (12h)	-4.932E-5	.000	-.161	-2.620	.009	NP	
Drain tube bleed (12h)	-9.499E-5	.000	-.121	-1.963	.051	NP	
Diuresis (12h)	-7.984E-5	.000	-.097	-1.566	.119	NP	

AVR: aortic valve replacement; BMI: body-mass-index; CABG: coronary artery bypass grafting; CPAP: continuous positive airway pressure; DHCA: deep hypothermic circulatory arrest; ECMO: extracorporeal membrane oxygenation; eGFR: estimated glomerular filtration rate; IABP: intra-aortic balloon-pump; LAA: left atrial appendage; LED: lupus erythematosus disseminatus; MPL: mitral valve plasty; MVR: mitral valve replacement; NSTEMI: non ST-elevation myocardila infarction; PCI: percutaneous coronary intervention; SLE: systemic lupus erythematosus; STEMI: ST-elevation myocardial infarction; TAVR: trancatheter aortic valve implantation; UAP: unstable angina pectoralis

Supplementary Table for review. Table shows multivariable logistic regression analysis of A) in-hospital adverse events; and B) 90d adverse events.

A)

Variables in the Equation

		B	S.E.	Wald	df	Sig.	Exp(B)	95% C.I.for EXP(B) Lower	Upper
Step 1[a]	female_male	-.395	.494	.639	1	.424	.674	.256	1.773
	age_calculated	.032	.020	2.646	1	.104	1.033	.993	1.073
	Log_deltaHIF	-.969	.367	6.987	1	.008	.379	.185	.778
	treatment_for_diabetes	-.178	.397	.201	1	.654	.837	.384	1.822
	treatment_for_hypertension	.182	.467	.153	1	.696	1.200	.481	2.994
	heart_failure	.749	.459	2.661	1	.103	2.114	.860	5.197
	pre.op_atrial_fibrillation	-.386	.443	.758	1	.384	.680	.285	1.621
	coronary_artery_disease	-.200	.394	.258	1	.611	.819	.378	1.772
	bmi	.020	.038	.275	1	.600	1.020	.947	1.099
	preop_eGFR	.013	.009	2.282	1	.131	1.013	.996	1.031
	Constant	-5.409	2.071	6.820	1	.009	.004		
Step 2[a]	female_male	-.385	.493	.609	1	.435	.681	.259	1.789
	age_calculated	.033	.020	2.812	1	.094	1.033	.994	1.074
	Log_deltaHIF	-.967	.366	6.972	1	.008	.380	.186	.779
	treatment_for_diabetes	-.167	.396	.178	1	.673	.846	.389	1.839
	heart_failure	.757	.458	2.725	1	.099	2.131	.868	5.235
	pre.op_atrial_fibrillation	-.380	.443	.736	1	.391	.684	.287	1.630
	coronary_artery_disease	-.163	.384	.180	1	.671	.850	.401	1.802
	bmi	.021	.038	.302	1	.583	1.021	.948	1.100
	preop_eGFR	.013	.009	2.198	1	.138	1.013	.996	1.030
	Constant	-5.347	2.059	6.743	1	.009	.005		
Step 3[a]	female_male	-.370	.491	.566	1	.452	.691	.264	1.809
	age_calculated	.032	.019	2.721	1	.099	1.033	.994	1.073
	Log_deltaHIF	-.960	.365	6.905	1	.009	.383	.187	.784
	heart_failure	.736	.455	2.614	1	.106	2.088	.855	5.099
	pre.op_atrial_fibrillation	-.363	.441	.676	1	.411	.696	.293	1.652
	coronary_artery_disease	-.201	.373	.291	1	.590	.818	.394	1.698
	bmi	.016	.036	.197	1	.657	1.016	.947	1.091
	preop_eGFR	.013	.009	2.144	1	.143	1.013	.996	1.030
	Constant	-5.165	1.999	6.678	1	.010	.006		
Step 4[a]	female_male	-.362	.491	.544	1	.461	.696	.266	1.822
	age_calculated	.031	.019	2.617	1	.106	1.032	.993	1.071
	Log_deltaHIF	-.950	.364	6.816	1	.009	.387	.190	.789
	heart_failure	.724	.454	2.537	1	.111	2.062	.846	5.026
	pre.op_atrial_fibrillation	-.354	.441	.645	1	.422	.702	.296	1.665
	coronary_artery_disease	-.184	.371	.246	1	.620	.832	.402	1.721
	preop_eGFR	.012	.009	2.097	1	.148	1.013	.996	1.030
	Constant	-4.643	1.596	8.460	1	.004	.010		
Step 5[a]	female_male	-.301	.475	.402	1	.526	.740	.292	1.878
	age_calculated	.028	.019	2.364	1	.124	1.029	.992	1.067
	Log_deltaHIF	-.965	.362	7.087	1	.008	.381	.187	.775
	heart_failure	.737	.454	2.636	1	.104	2.090	.858	5.087
	pre.op_atrial_fibrillation	-.319	.436	.534	1	.465	.727	.309	1.709
	preop_eGFR	.012	.009	2.126	1	.145	1.013	.996	1.030
	Constant	-4.616	1.596	8.364	1	.004	.010		
Step 6[a]	age_calculated	.027	.018	2.174	1	.140	1.027	.991	1.065
	Log_deltaHIF	-.984	.361	7.443	1	.006	.374	.184	.758
	heart_failure	.732	.453	2.615	1	.106	2.080	.856	5.053
	pre.op_atrial_fibrillation	-.272	.429	.403	1	.525	.761	.328	1.765
	preop_eGFR	.014	.008	2.601	1	.107	1.014	.997	1.030
	Constant	-4.655	1.586	8.612	1	.003	.010		
Step 7[a]	age_calculated	.025	.018	1.921	1	.166	1.025	.990	1.062
	Log_deltaHIF	-.960	.359	7.170	1	.007	.383	.189	.773
	heart_failure	.656	.437	2.258	1	.133	1.928	.819	4.538
	preop_eGFR	.014	.008	2.577	1	.108	1.014	.997	1.030
	Constant	-4.550	1.573	8.366	1	.004	.011		
Step 8[a]	Log_deltaHIF	-.965	.359	7.220	1	.007	.381	.188	.770
	heart_failure	.641	.436	2.167	1	.141	1.899	.809	4.458
	preop_eGFR	.009	.008	1.330	1	.249	1.009	.994	1.024
	Constant	-2.569	.637	16.259	1	<.001	.077		
Step 9[a]	Log_deltaHIF	-.932	.355	6.882	1	.009	.394	.196	.790
	heart_failure	.557	.428	1.695	1	.193	1.746	.755	4.039
	Constant	-1.888	.209	81.837	1	<.001	.151		
Step 10[a]	Log_deltaHIF	-.907	.354	6.578	1	.010	.404	.202	.807
	Constant	-1.792	.190	88.872	1	<.001	.167		

a. Variable(s) entered on step 1: female_male, age_calculated, Log_deltaHIF, treatment_for_diabetes, treatment_for_hypertension, heart_failure, pre.op_atrial_fibrillation, coronary_artery_disease, bmi, preop_eGFR.

B)

Variables in the Equation

		B	S.E.	Wald	df	Sig.	Exp(B)	95% C.I.for EXP(B)	
								Lower	Upper
Step 1[a]	female_male	-.177	.435	.166	1	.684	.838	.357	1.966
	age_calculated	.028	.018	2.276	1	.131	1.028	.992	1.066
	Log_deltaHIF	-.761	.351	4.689	1	.030	.467	.235	.930
	treatment_for_diabetes	.009	.366	.001	1	.981	1.009	.493	2.065
	treatment_for_hypertension	.357	.454	.620	1	.431	1.430	.587	3.481
	heart_failure	.731	.426	2.943	1	.086	2.078	.901	4.792
	pre.op_atrial_fibrillation	-.308	.410	.562	1	.453	.735	.329	1.643
	coronary_artery_disease	-.269	.368	.531	1	.466	.764	.371	1.574
	bmi	.029	.035	.703	1	.402	1.030	.961	1.103
	preop_eGFR	.007	.008	.678	1	.410	1.007	.991	1.023
	Constant	-4.861	1.922	6.393	1	.011	.008		
Step 2[a]	female_male	-.178	.434	.168	1	.682	.837	.357	1.960
	age_calculated	.028	.018	2.291	1	.130	1.028	.992	1.066
	Log_deltaHIF	-.761	.351	4.703	1	.030	.467	.235	.929
	treatment_for_hypertension	.358	.453	.624	1	.430	1.431	.588	3.479
	heart_failure	.732	.424	2.984	1	.084	2.080	.906	4.775
	pre.op_atrial_fibrillation	-.309	.408	.572	1	.450	.734	.330	1.634
	coronary_artery_disease	-.267	.359	.552	1	.457	.766	.379	1.547
	bmi	.030	.033	.781	1	.377	1.030	.965	1.100
	preop_eGFR	.007	.008	.682	1	.409	1.007	.991	1.023
	Constant	-4.869	1.888	6.651	1	.010	.008		
Step 3[a]	age_calculated	.026	.018	2.151	1	.142	1.027	.991	1.064
	Log_deltaHIF	-.776	.349	4.947	1	.026	.460	.232	.912
	treatment_for_hypertension	.351	.453	.600	1	.438	1.421	.584	3.454
	heart_failure	.736	.424	3.018	1	.082	2.087	.910	4.789
	pre.op_atrial_fibrillation	-.277	.401	.477	1	.490	.758	.346	1.663
	coronary_artery_disease	-.227	.346	.431	1	.512	.797	.404	1.571
	bmi	.029	.033	.758	1	.384	1.030	.964	1.099
	preop_eGFR	.007	.008	.856	1	.355	1.007	.992	1.023
	Constant	-4.874	1.884	6.694	1	.010	.008		
Step 4[a]	age_calculated	.024	.018	1.854	1	.173	1.024	.989	1.060
	Log_deltaHIF	-.786	.348	5.101	1	.024	.456	.231	.901
	treatment_for_hypertension	.279	.438	.406	1	.524	1.322	.560	3.121
	heart_failure	.750	.423	3.141	1	.076	2.118	.924	4.856
	pre.op_atrial_fibrillation	-.244	.399	.376	1	.540	.783	.359	1.710
	bmi	.028	.033	.695	1	.404	1.028	.963	1.098
	preop_eGFR	.007	.008	.789	1	.374	1.007	.992	1.023
	Constant	-4.750	1.866	6.476	1	.011	.009		
Step 5[a]	age_calculated	.022	.017	1.623	1	.203	1.022	.988	1.057
	Log_deltaHIF	-.764	.346	4.871	1	.027	.466	.236	.918
	treatment_for_hypertension	.283	.437	.417	1	.518	1.327	.563	3.127
	heart_failure	.684	.409	2.797	1	.094	1.982	.889	4.421
	bmi	.027	.033	.659	1	.417	1.027	.963	1.096
	preop_eGFR	.007	.008	.774	1	.379	1.007	.992	1.023
	Constant	-4.625	1.845	6.285	1	.012	.010		
Step 6[a]	age_calculated	.024	.017	2.004	1	.157	1.024	.991	1.059
	Log_deltaHIF	-.758	.345	4.824	1	.028	.469	.238	.922
	heart_failure	.689	.409	2.847	1	.092	1.992	.895	4.438
	bmi	.029	.033	.780	1	.377	1.030	.965	1.098
	preop_eGFR	.007	.008	.692	1	.405	1.007	.991	1.022
	Constant	-4.554	1.831	6.182	1	.013	.011		
Step 7[a]	age_calculated	.019	.015	1.465	1	.226	1.019	.988	1.050
	Log_deltaHIF	-.740	.343	4.650	1	.031	.477	.243	.935
	heart_failure	.631	.402	2.460	1	.117	1.879	.854	4.131
	bmi	.028	.033	.709	1	.400	1.028	.964	1.097
	Constant	-3.677	1.469	6.264	1	.012	.025		
Step 8[a]	age_calculated	.018	.015	1.353	1	.245	1.018	.988	1.049
	Log_deltaHIF	-.726	.341	4.531	1	.033	.484	.248	.944
	heart_failure	.609	.401	2.311	1	.128	1.839	.838	4.033
	Constant	-2.824	1.039	7.394	1	.007	.059		
Step 9[a]	Log_deltaHIF	-.739	.342	4.663	1	.031	.477	.244	.934
	heart_failure	.633	.400	2.507	1	.113	1.883	.860	4.124
	Constant	-1.650	.192	73.721	1	<.001	.192		
Step 10[a]	Log_deltaHIF	-.712	.341	4.366	1	.037	.491	.252	.957
	Constant	-1.541	.174	78.085	1	<.001	.214		

a. Variable(s) entered on step 1: female_male, age_calculated, Log_deltaHIF, treatment_for_diabetes, treatment_for_hypertension, heart_failure, pre.op_atrial_fibrillation, coronary_artery_disease, bmi, preop_eGFR.
